# Programmed cell death ligands expression in phaeochromocytomas and paragangliomas: Relationship with the hypoxic response, immune evasion and malignant behavior

**DOI:** 10.1080/2162402X.2017.1358332

**Published:** 2017-08-04

**Authors:** David J. Pinato, James R. Black, Sebastian Trousil, Roberto E. Dina, Pritesh Trivedi, Francesco A. Mauri, Rohini Sharma

**Affiliations:** a Department of Surgery & Cancer, Imperial College London, Hammersmith Hospital, Du Cane Road, W120HS London, UK; b Cutaneous Biology Research Centre, Massachusetts General Hospital and Harvard Medical School, Charlestown, MA, USA; c Department of Histopathology, Imperial College London, Hammersmith Hospital, Du Cane Road, W120HS London, UK

**Keywords:** Phaeochromocytoma, Paraganglioma, PD-L1, PD-L2, prognosis

## Abstract

The hypoxic response underlies the pathogenesis and malignant behavior of PCC/PGL. Regulation of PD-1 receptor-ligand signaling, a therapeutically actionable driver of the anti-tumor immune response, is a hypoxic-driven trait across malignancies. We evaluated the prognostic role of PD ligands in association with biomarkers of hypoxia and angiogenesis in patients with PCC/PGL.

Tissue microarrays sections including consecutive cases diagnosed between 1983–2011 were stained for PD-L1 and 2, hypoxia inducible factor 1a (Hif-1a), Carbonic Anhydrase IX (CaIX), Vascular Endothelial Growth Factor-A (VEGF-A). We explored the biologic significance of PD ligands expression using gene set enrichment analysis (GSEA) on The Cancer Genome Atlas (TCGA) for PCC/PGL (n = 184).

In total, 100 patients, 10% malignant, 64% PCC, 29% familial with median tumor size of 4.7 cm (range 1–14) were included. Median follow-up was 4.7 y. We found PD-L1 expression in 18% of PCC/PGL, which was independent of adverse pathological features including capsular (CI), vascular invasion (VI), necrosis (N) and expression of biomarkers of hypoxia. PD-L2 expression (16%) strongly correlated with CI, VI, N and malignant behavior (p < 0.05) and was associated with stronger Hif-1a and CaIX immunolabeling (p < 0.01). PD-L2 was predictive of shorter survival (162 versus 309 months, HR 3.1 95%CI 1.1–9.2, p = 0.02). GSEA on TGCA samples confirmed enrichment of transcripts involved in hypoxia and anti-cancer immunity.

We report for the first time PD ligands expression in PCC/PGL with a distinctive prognostic, clinico-pathologic and immuno-biologic role. These findings support a potential therapeutic role for PD-1/PD-L1 targeted checkpoint inhibitors in these tumors.

**KEY MESSAGE**

The molecular mechanisms underlying immune evasion in malignant phaeochromocytomas and paragangliomas (PCC/PGL) are poorly understood. This study demonstrates for the first time a distinctive immune-biologic and prognostic role of programmed death ligands 1 and 2 (PD-L1, PD-L2), two actionable drivers of the anti-cancer immune response. RNA-sequencing of tumor tissues reveals enrichment of transcripts relating to hypoxia and immune-exhaustion to explain the adverse clinical course observed in PD-L2 overexpressing tumors. These findings provide a rationale for the development of anti PD-1 therapies in malignant PCC/PGL.

## Introduction

Phaeochromocytomas (PCCs) and paragangliomas (PGLs) are rare catecholamine-producing neuroendocrine tumors (NETs) deriving from adrenal (PCC) or extra-adrenal (PGL) chromaffin tissue.^1^

Surgical resection, the mainstay of treatment in PCC/PGL, is curative in most patients. However, almost 20% of tumors are malignant with the potential to recur and spread systemically, with negative implications for prognostic outlook.^2^

Treatment options for metastatic PCC/PGL are limited to radionuclide^3,4^ and cytotoxic chemotherapy, the efficacy of which mostly relies on retrospective reports of small numbers of patients.^5^ Systemic chemotherapy may control disease progression^6^ but does not prolong survival.^7^ Because of these major shortcomings in managing metastatic PCC/PGL, 5-year survival rates are <50%^8^ and inferior to those seen in other metastatic NETs.^9^

Therapeutic inhibition of the programmed cell death (PD-1) receptor-ligand immune checkpoint has recently revolutionised the systemic treatment of malignancy.^10^

By antagonising the immune-suppressive interaction between PD-1, a T-cell co-inhibitory receptor, and its ligand PD-L1, therapeutic antibodies against this pathway can restore an efficacious anti-tumor immune response, manifest as durable clinical responses in a proportion, but not all patients.^11^

Identification of biologic predictors of response is therefore a priority in attempting to develop immunotherapy, more so for rare malignancies where enrichment of molecularly phenotyped patient populations with enhanced potential to respond to treatment is highly desirable in clinical trial design.

PD-L1 expression by immunohistochemistry has been advocated as a putative predictive correlate of response to anti-PD-1 therapies and incorporated as a biomarker in clinical studies since initial early-phase trials of immune-checkpoint inhibitors.^12^

While the stratifying potential of PD-L1 is imperfect because of significant clinical responses having been observed in PD-L1 negative tumors,^13^ its use has expanded in certain malignancies where prospective data has correlated high PD-L1 expression with longer progression-free and overall survival following immune-checkpoint inhibition.^14^

More recently, tumor cell expression of PD-L2, an important modulator of T-helper 2 cell responses^15^ has been independently correlated with the efficacy of PD-1 axis targeted inhibitors suggesting that the diversity in PD ligands expression might contribute to the heterogeneity of clinical responses to checkpoint inhibitors.^16^

Biologically, elevated PD-L1 expression within tumor cells or the surrounding microenvironment is dynamic as a result of various molecular events including hypoxia^17^ and similar correlations have been highlighted for PD-L2.^18^ We have previously shown that activation of hypoxia inducible factor 1α (Hif-1α) is a key molecular hallmark in the metastatic progression of PCC/PGL,^19^ a disease where constitutive Hif-1α stabilization secondary to mitochondrial dysfunction, termed “pseudo-hypoxia,” is a key pathophysiological trait.^20^

In a disease where the anti-cancer immune response has remained largely unexplored, we hypothesized that activation of the hypoxic response might promote cancer-specific immune-tolerance through expression of PD ligands and thus facilitate malignant progression in PCC/PGL. To test this hypothesis, and attempt to qualify novel targets for immunotherapy, we evaluated the prevalence and clinico-pathologic significance of PD-L1 and PD-L2 expression in a well-annotated series of PCC/PGL.

## Materials and methods

### Patient characteristics

We included 100 consecutive PCC/PGL patients who underwent surgical treatment at Imperial College, a tertiary referral center with a specialist NET board, from 1983–2011. Haematoxylin and eosin (H&E) slides were reviewed to identify relevant pathological features and archival paraffin-embedded samples were retrieved.

Clinico-pathologic data, including tumor characteristics and survival, was gathered from medical records. Follow-up was updated prospectively and censored in November 2016. Malignant behavior was defined as detection of metastasis to lymph nodes or distant sites either at diagnosis or during subsequent review. Ethical approval was granted by the Imperial College Tissue Bank (Ref. R14066–2A).

### Tissue microarrays (TMAs) and immunohistochemistry (IHC)

TMA blocks were prepared as described previously.^21^ All the samples utilised were surgical specimens. No biopsies were included. One mm tumor cores were sampled in triplicate using a MTA-1 Manual Tissue Microarrayer (Beecher Instruments, USA). IHC staining was performed on 5μm sections using the Leica Bond RX stainer (Leica, Buffalo, IL).

The primary antibodies anti-PD-L1 (Clone E1L3N; Cell Signaling Cat. Nr. 13684), anti-PD-L2 (Sigma Aldrich, Cat. Nr. 3500395) were incubated overnight at the concentration 1:100 and 1:300, respectively as described before.^22^ IHC staining for biomarkers of hypoxia/angiogenesis including VEGF-A (Cat. Nr. Sc- 152; Santa Cruz Biotechnology Inc., Santa Cruz, CA, USA), Hif-1α (Cat. Nr. Ab8366 AbCam, Cambridge, UK) and CaIX (Cat. Nr. NB100–417, Novus Biologicals, Cambridge, UK) followed described previously methodology.^21^ Sections were incubated for 1 hour at room temperature with the secondary antibody and processed using the Polymer-HRP system (BioGenex, Fremont, CA, USA), developed in diaminobenzidine and counterstained in Mayer's Haematoxylin.

Biomarker expression was scored independently by 2 histopathologists (FAM, RED) using a semi-quantitative immuno-histoscore (IHS). For clinicopathologic correlation, continuous IHS ranging from 0–300 were categorised around the median of the distribution. Accounting for the focal pattern of PD-L1 expression cases displaying moderate intensity of signal in ≥5% of tumor cells were considered positive.^22^

### Gene set enrichment analysis (GSEA)

To characterize signaling pathways associated with PD-ligands expression we performed GSEA using RNA-sequencing data from the TCGA cohort of PCC/PGL (n = 184). PD-L1 (CD274) and PD-L2 (PDCD1LG2) were used as phenotypic labels. Analyzed gene sets included validated hallmark signatures derived from the MSigDB database to reflect the hypoxic response and a variety of transcriptional programs involved in anti-cancer immunity including the inflammatory response, the IL-6/JAK/STAT3 pathway, IL-2/STAT5 pathway, and signatures relating to Interferon-γ, IFN-α, Tumor Necrosis Factor a (TNF-α) and Transforming-Growth Factor β (TGF-β). We evaluated enrichment in phenotype both in positive and negative correlation with PD-L1 and PD-L2.

Using cBioportal (http://www.cbioportal.org, accessed 23/03/2017) we examined the 20 genes displaying the strongest positive correlation with PD ligands by Pearson's correlation index. Using oncoprint analysis we evaluated PD ligands expression in relationship to the presence of mutations in key susceptibility loci for PCC/PGL including Succinate-Dehydrogenase (SDH) subunits A-D, Von-Hippel Lindau (VHL), Neurofibromatosis-1 (NF-1) and c-RET.

### Statistical analysis

Pearson's χ^2^ or Fisher's exact tests (2-tailed) were used to test for any significant associations between variables as indicated. A *p* value of <0.05 was used as a threshold for statistical significance. Kaplan-Meier curves were used for survival analyses. IBM SPSS version 23 (SPSS inc., Chicago, IL, USA) was used for all statistical analysis.

## Results

### Patient characteristics

The clinico-pathologic features of the patient cohort are displayed in (Table 1). Patients were followed up over a median period of 4.7 y (range 6 months – 34 years). Median age at diagnosis was 40 y. Malignant disease was identified radiologically or histologically in 10 patients as either *de novo* metastatic presentation (n = 3) or systemic recurrence during follow-up (n = 7). Twenty-nine patients had syndromic PCC/PGL based on germline DNA testing: the most prevalent mutation was in the RET proto-oncogene (n = 13) followed by mutation in one of the SDH subunits (n = 11), VHL (n = 3), NF-1 (n = 2). The most prevalent genotype in malignant cases was mutation in SDH-B (n = 2) followed by VHL (n = 1) and NF-1 (n = 1).

**Table 1.** Clinical characteristics of patients with PCC/PGL.

Baseline characteristic	n = 100
Gender	
Male	46
Female	54
Age (years)	
<40	50
≥ 40	50
Disease Site	
PCC monolateral	59
PCC bilateral	5
PGL (Extra-adrenal)	36
Maximum tumor diameter	
< 5 cm	70
≥ 5 cm	30
Behavior	
Benign	90
Malignant	10
Catecholamine secretion	
Noradrenaline	54
Adrenaline	28
Dopamine	15
Silent	20
Missing	13
Tumor Necrosis	
Absent	76
Present	16
Capsular Invasion	
Absent	79
Present	13
Vascular Invasion	
Absent	84
Present	8
Genotype (germline mutations)	
SDH-B	9
SDH-D	2
NF-1	2
VHL	3
RET	12

All patients had surgery as the principal treatment of their tumor. Six patients with metastatic disease received radiotherapy, 2 received chemotherapy, and 4 received Iodine-131-meta-iodobenzylguanidine therapy.

### PD ligands expression in PCCs/PGLs

**Figure 1.**
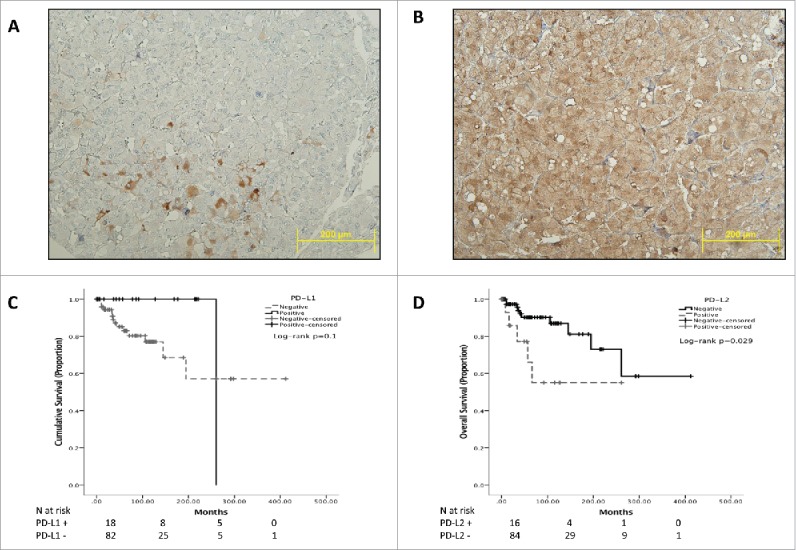
Representative sections of PCC/PGL TMA cores. Panel (A)shows focal expression of PD-L1 in a malignant case and the diffuse pattern of immunopositivity typical of PD-L2 immunostaining (Panel B). Panel (B) highlights the specificity of PD-L2 staining for PCC/PGL cells where sustentacular cells are negative for PD-L2 expression. Panels C and D show Kaplan-Meier curves describing the overall survival of patients with PCC/PGL categorized according to PD-L1 (Panel C) and PD-L2 (Panel D).

In total, 18 cases stained positively for PD-L1 according to the pre-specified cut-off. PD-L1 staining was focal, with a cytoplasmic and membranous pattern of immunopositivity as shown in Fig. 1A. The prevalence of PD-L1 immunopositivity was 40% in malignant (4/10) and 15% (14/90) in benign cases (Fisher's Exact test p = 0.08). There was no significant association between PD-L1 positivity and any of the salient clinico-pathologic features including type of underlying germline mutation, tumor size, tumor localization (adrenal vs. extra-adrenal).

We identified PD-L2 expression in 16 cases, where the pattern of immunopositivity observed was cytoplasmic and diffuse (Fig. 1B). The median PD-L2 IHS was 100 (mean 146, range 60–300). PD-L2 expression was positively associated with malignant behavior. Prevalence of PD-L2 expression was 50% in malignant cases (5/10) compared with 12% in benign specimens (11/90, Pearson Chi-square p = 0.002). The proportion of PD-L2 positivity was higher in vasculo-invasive PCC/PGL (5/7 cases, 71% vs. 11/84, 13%, p < 0.001). Similarly, we found a higher rate of PD-L2 immunopositivity in cases displaying capsular invasion (6/13, 46% vs. 10/79, 13%, p = 0.003) and necrosis (7/16, 44% vs. 9/76, 12%, p = 0.002). We found evidence of PD-L1 and PD-L2 co-immuno expression in 3 cases, with a positive association with malignancy (1/89 cases vs. 2/10, Fisher Exact test p = 0.02).

A total of 26 samples showed high CaIX expression levels (median IHS 200, mean 178, range 75–275) whereas VEGF-A was overexpressed in 64 (median IHS 200, mean 178, range 75–275) and Hif-1α in 35 (median IHS 120, mean 107, range 0–300) (Supplementary Fig. S1).

**Figure 2.**
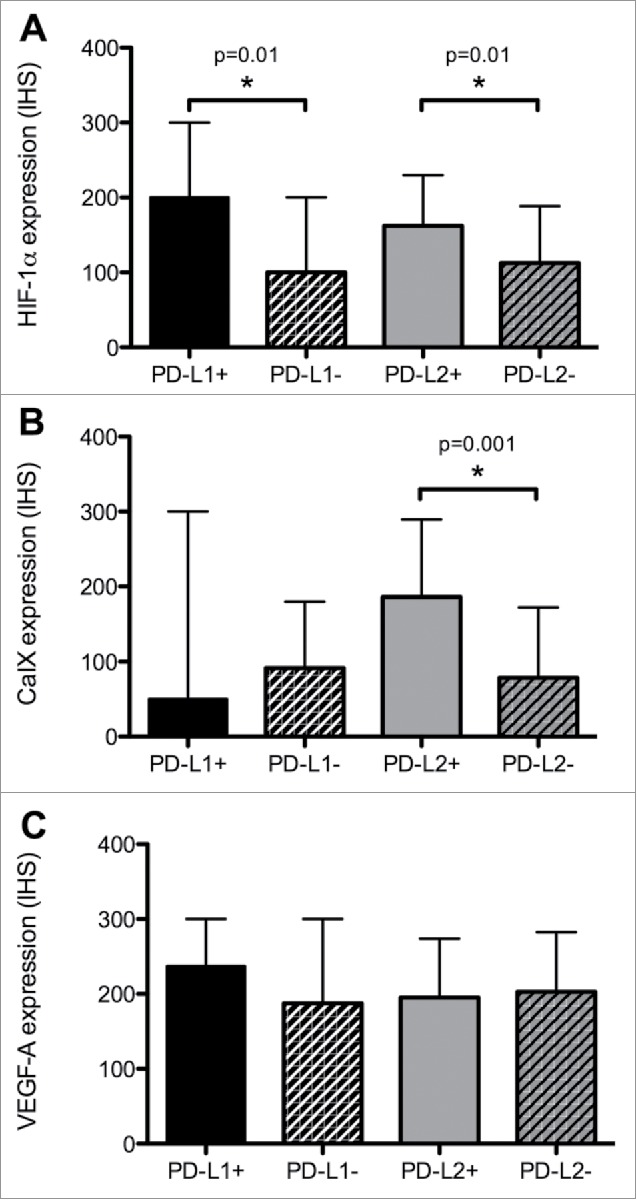
The relationship between PD ligands and the expression of Hif-1α (Panel A), CaIX (Panel B) and VEGF-A (Panel C). Expression of candidate biomarkers is quantified using an immuno-histoscore (IHS). Median values (+/− interquartile ranges) are presented. An asterisk (*) highlights a statistically significant difference in median IHS values resulting from a Mann-Whitney U test.

We evaluated the relationship between the expression of biomarkers of hypoxia and angiogenesis and PD ligands. As shown in Fig. 2A–B, PD-L1-positive PCC/PGL were characterized by higher Hif-1α (median IHS 200 vs. 100, Mann Whitney U = 396.0, p = 0.01) but not CaIX.

Similarly, PD-L2 overexpressing tumors had a higher proportion of Hif-1α (median IHS 166 vs. 100, Mann Whitney U = 407.0, p = 0.01) and CaIX overexpression (median IHS 33 vs. 200, Mann Whitney U = 280.0, p < 0.001). We found no relationship between PD ligands and VEGF-A expression (Fig. 2C).

### PD-L1 and PD-L2 expression: relationship with malignancy and survival

We evaluated the prognostic value of PD-L1 and PD-L2 expression in the whole study population where mean OS was 25 y (95% CI 12–29 years) and median OS was not reached. OS was significantly shorter in malignant cases where mean OS was 7.2 y (2.2–12.3 years) and median OS was 3.6 y (95%CI 1.2–6 years, Log-rank p < 0.001).

The expression of PD-L1 was not predictive of OS in the entire patient population, with PD-L1 positive cases not differing from PD-L1 negative counterparts (Fig. 1C). When considering PD-L2 expression, we found that in patients overexpressing PD-L2 OS was significantly shorter with a median of 13.5 y (95%CI 8–19 years) compared with 25 y of patients with PD-L2 negative PCC/PGL (95%CI 20–30 years, Log-rank p = 0.029, Fig. 1D).

Other prognostic factors included presence of vascular invasion, capsular invasion and necrosis (Supplementary Fig. S2, Supplementary Table S1).

### Differential relationship between PD-L1, PD-L2 and hypoxia: validation in TCGA data set

**Figure 3.**
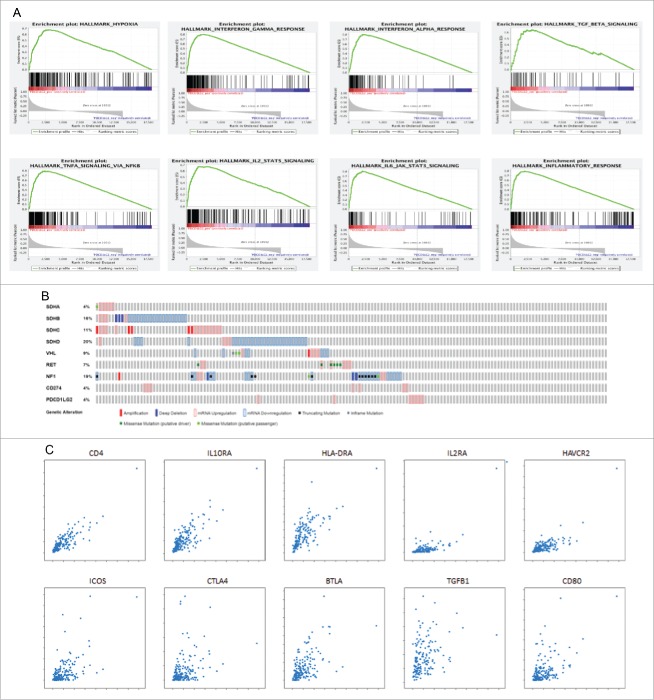
Panel A illustrates the enrichment plots for 8 hallmark signatures positively correlating with PD-L2 (PDCD1LG2) expression in the PCC/PGL TCGA data set (n = 184). Panel B illustrates the relationship between PD-L1 and 2 expression and functionally relevant genomic alterations in 6 major susceptibility loci for familial PCC/PGL (SDH subunits A-D, VHL, NF-1 and RET). Panel C illustrates linear regression analysis correlating PD-L2 expression with a panel of genes reflecting immune-exhausted T cell responses including CD4, IL10RA, HLA-DRA, IL2RA, HAVCR2 (Tim-3), ICOS, CTLA-4, BTLA, TGFB1, CD80.

We used the PCC/PGL TCGA data set to validate the relationship between PD-L1 and 2 expression and hallmarks of hypoxia. Consistent with our immunohistochemical findings, we found enrichment of transcripts involved in the hypoxic response in relationship with PD-L2 but not PD-L1 expression (Fig. 3A, Supplementary Tables S2 and S3).

We addressed mutational status in key susceptibility loci for PCC/PGL as a potential confounder, demonstrating a lack of enrichment for PD-ligand overexpression in tumor samples harbouring mutations in any of the tested loci (Fig. 3B).

### Immuno-biologic qualification of PD-L1 and PD-L2 in PCC/PGL

Using GSEA we found PD-L2 overexpressing PCC/PGL to be strongly enriched in transcriptional programs innate and adaptive anti-cancer immunity (Fig. 3A). Conversely, none of the gene sets were significantly enriched in PD-L1 overexpressing PCC/PGL, where we found a trend toward a positive association with a subset of 96 genes involved in protein secretion (nominal p = 0.03, FDR = 1) (Supplementary Table S1).

We confirmed differential gene expression profiles in correlation with PD ligands with a preferential co-expression of immune-inflammatory genes in relationship with PD-L2 as opposed to PD-L1 (Table 2). Given CD4 emerged as the strongest correlate to PD-L2 expression (Pearson's R 0.85, Fig. 3C) we phenotypically characterized T-cell enrichment revealing a significantly positive association in genes characterizing an exhausted immune response as shown in Fig. 3C.

**Table 2.** Gene transcripts that are most strongly co-expressed in PD-L1 and PD-L2 overexpressing PCC/PGLs from the TCGA data set (n = 184).

PD-L1			PD-L2		
Gene Symbol	Pearson Score	Spearman Score	Gene Symbol	Pearson Score	Spearman Score
DNAJB14	0.75	0.75	CD4	0.85	0.76
MAN2A1	0.7	0.62	ARHGAP30	0.85	0.74
PCDHAC2	0.7	0.74	CTSS	0.82	0.75
PCDHAC1	0.68	0.72	FERMT3	0.82	0.71
ATP2B4	0.67	0.59	MS4A4A	0.82	0.78
IL6ST	0.67	0.66	AOAH	0.81	0.73
OXCT1	0.67	0.65	CASP1	0.81	0.81
G3BP2	0.67	0.71	IL10RA	0.81	0.72
MID2	0.67	0.7	PLEK	0.81	0.7
THSD7A	0.67	0.67	SASH3	0.81	0.72
PTPRJ	0.66	0.57	CPVL	0.81	0.74
TBC1D9	0.66	0.6	C1QC	0.8	0.72
SPOPL	0.65	0.71	IL12RB1	0.8	0.66
CERS6	0.65	0.69	MS4A6A	0.8	0.74
STT3B	0.64	0.6	CTSC	0.79	0.73
RNASEH1	0.64	0.6	FPR3	0.79	0.66
ERAP1	0.64	0.59	HCK	0.79	0.73
ELMOD2	0.64	0.69	SECTM1	0.79	0.66
ZNF106	0.64	0.73	SIGLEC1	0.79	0.67

## Discussion

The lack of effective therapies is a highly unmet need in PCC/PGLs, a rare subtype of NETs where prediction of malignant behavior has so far been elusive and novel molecular targets are at the focus of intense research efforts.^23^

Previous work has highlighted several molecular differences between benign and malignant PCC/PGL. Activation of the hypoxic response mediated by stabilization of Hifs is a highly prevalent molecular mechanism^24^ contributing to malignant progression of PCC/PGL.^25^

Immune-evasion is a key hallmark of cancer progression and expression of PD ligands is a common mechanism through which tumor cells counteract immunologic clearance by negative regulation of PD-1-expressing lymphocyte populations. The presence of hypoxia-response elements (HRE) within the PD-L1 promoter region has highlighted a therapeutically attractive link between hypoxia/angiogenesis and anti-tumor immune suppression,^26^ with similar mechanisms being implied in the regulation of PD-L2 expression.^27^

In our study we document for the first time differential regulation of PD ligands in PCC/PGLs, where half of the malignant cases express at least one of the PD ligands to support their potential contribution in shaping the immune-tolerogenic environment.

Interestingly, while PD-L1 did not correlate with patients' survival, PD-L2 overexpression identified a subset of patients with poorer prognosis following radical resection. The negative impact of PD-L2 on patients' prognosis is further corroborated by the relationship between PD-L2 status and several histopathologic hallmarks of a more aggressive disease course including vascular, capsular invasion and necrosis – all of which were confirmed as predictors of poor outcome in our patient series.

Furthermore, in line with previously published studies in other malignancies, we found a positive correlation between Hif-1α and PD ligands expression, stronger for PD-L2 over PD-L1, a finding that we independently validated using the TCGA RNA-Seq data set.

Interestingly, PD ligands expression was not influenced by germline mutations neither in our case series nor in the TGCA data set to suggest that pseudo-hypoxic signals might not be the only drivers of PD ligands expression.^28^

This is further strengthened by the non-uniform relationship we found between PD ligands and the expression of CaIX or VEGF-A, 2 renown targets of Hif-1α, implying that the regulation of PD-ligands in the context hypoxic/pro-angiogenic signals might involve other molecular actors.^29^

Analysis of RNA-seq data portrayed more comprehensively the biologic heterogeneity characterizing the relationship between PD ligands and tumor hypoxia.

In fact, when we considered a broader subset of 200 genes involved in the hypoxic response, PD-L2 upregulation strikingly emerged as a stronger and more substantial determinant of tumor hypoxia than PD-L1, to suggest a potential mechanistic relationship between hypoxia and PD-L2-mediated anti-tumor immune control. When evaluating individual genes in more detail, we found that, while PD-L1 expression was predominantly associated with genes involved in endoplasmic reticulum trafficking and granule secretion such as DNAJB14, MAN2A1, PCDHAC1–2, the expression of PD-L2 was strongly and consistently associated with several transcripts involved in innate and adaptive immunity including CD4, IL10RA, IL12RB1 and C1QC to suggest inherent differences in their immunobiologic role (Table 2).

In several oncological indications where PD-1-targeted checkpoint inhibitors have been eventually proven effective in prospective trials, the clinical efficacy of these therapies was anticipated by prior clinicopathologic studies showing a relationship between PD ligands and clinical outcome.^12^ While the likelihood of response to immunotherapy cannot be directly inferred from our data, we provide evidence of differential regulation of PD-L1 and PD-L2 in our clinical samples, with diverse ramifications in terms of their clinico-pathologic significance and predictive role in influencing patients' survival.

Interestingly, our data suggest PD-L2 to have a more predominant role than PD-L1 in shaping the immune-tolerogenic environment, given the highly significant association with key pathways involved in innate, adaptive immunity and inflammation.

The enrichment of immunologic markers suggestive of an exhausted immune response including TGF-β-related signatures and several regulatory T-cell markers (IL10RA, IL10RB, HLA-DRA) as well as immune checkpoints (Tim-3, BTLA, CTLA-4, CD80, ICOS) provides strong immunobiologic foundations to justify the relationship between PD-L2 expression and poorer prognosis in patients with PCC/PGL.

Our results therefore provide a valuable clinical rationale for the development of immune checkpoint inhibitors in a subset PCC/PGL where anti-tumor immune exhaustion is a key prognostic trait and suggests PD-L2 expression as a putative stratifying biomarker in prospective clinical studies.

We acknowledge the relatively small sample size and the rarity of malignant cases as a limitation to our study, which is unfortunately shared with most of the published work in this field.

Nonetheless, taken together, our findings support the existence of a subset of PCC/PGL with biologic bases for PD-1–mediated tumor immune evasion and highlights a potential role for immunotherapy targeting the PD-1 ligand-receptor axis. The relationship between hypoxia, malignancy and anti-tumor immune control should be further explored in prospective studies with the aim of facilitating the development of immunotherapy in this otherwise neglected disease area.

## Supplementary Material

Supplementary_materials.doc

## References

[1] Gimm O, DeMicco C, Perren A, Giammarile F, Walz MK, Brunaud L Malignant pheochromocytomas and paragangliomas: a diagnostic challenge. Langenbecks Arch Surg. 2012;397:155-77. doi:10.1007/s00423-011-0880-x. PMID:22124609 22124609

[2] Manger WM, Eisenhofer G Pheochromocytoma: diagnosis and management update. Curr Hypertens Rep. 2004;6:477-84. doi:10.1007/s11906-004-0044-2. PMID:15527694 15527694

[3] Gonias S, Goldsby R, Matthay KK, Hawkins R, Price D, Huberty J, Damon L, Linker C, Sznewajs A, Shiboski S et al. Phase II study of high-dose [131I]metaiodobenzylguanidine therapy for patients with metastatic pheochromocytoma and paraganglioma. J Clin Oncol. 2009;27:4162-8. doi:10.1200/JCO.2008.21.3496. PMID:19636009 19636009 PMC2734428

[4] Pinato DJ, Black JR, Ramaswami R, Tan TM, Adjogatse D, Sharma R Peptide receptor radionuclide therapy for metastatic paragangliomas. Med Oncol. 2016;33:47. doi:10.1007/s12032-016-0737-9. PMID:27059363 27059363

[5] Ayala-Ramirez M, Feng L, Habra MA, Rich T, Dickson PV, Perrier N, Phan A, Waguespack S, Patel S, Jimenez C Clinical benefits of systemic chemotherapy for patients with metastatic pheochromocytomas or sympathetic extra-adrenal paragangliomas: insights from the largest single-institutional experience. Cancer. 2012;118:2804-12. doi:10.1002/cncr.26577. PMID:22006217 22006217 PMC3882190

[6] Niemeijer ND, Alblas G, van Hulsteijn LT, Dekkers OM, Corssmit EP Chemotherapy with cyclophosphamide, vincristine and dacarbazine for malignant paraganglioma and pheochromocytoma: systematic review and meta-analysis. Clin Endocrinol (Oxf). 2014;81:642-51. doi:10.1111/cen.12542. PMID:25041164 25041164

[7] Tanabe A, Naruse M, Nomura K, Tsuiki M, Tsumagari A, Ichihara A Combination chemotherapy with cyclophosphamide, vincristine, and dacarbazine in patients with malignant pheochromocytoma and paraganglioma. Horm Cancer. 2013;4:103-10. doi:10.1007/s12672-013-0133-2. PMID:23361939 23361939 PMC10358011

[8] Hescot S, Leboulleux S, Amar L, Vezzosi D, Borget I, Bournaud-Salinas C, de la Fouchardiere C, Libé R, Do Cao C, Niccoli P et al. One-year progression-free survival of therapy-naive patients with malignant pheochromocytoma and paraganglioma. J Clin Endocrinol Metab. 2013;98:4006-12. doi:10.1210/jc.2013-1907. PMID:23884775 23884775

[9] Yao JC, Hassan M, Phan A, Dagohoy C, Leary C, Mares JE, Abdalla EK, Fleming JB, Vauthey JN, Rashid A et al. One hundred years after “carcinoid:” epidemiology of and prognostic factors for neuroendocrine tumors in 35,825 cases in the United States. J Clin Oncol. 2008;26:3063-72. doi:10.1200/JCO.2007.15.4377. PMID:18565894 18565894

[10] La-Beck NM, Jean GW, Huynh C, Alzghari SK, Lowe DB Immune Checkpoint Inhibitors: New Insights and Current Place in Cancer Therapy. Pharmacotherapy. 2015;35:963-76. doi:10.1002/phar.1643. PMID:26497482 26497482

[11] Muenst S, Soysal SD, Tzankov A, Hoeller S The PD-1/PD-L1 pathway: biological background and clinical relevance of an emerging treatment target in immunotherapy. Expert Opin Ther Targets. 2015;19:201-11. doi:10.1517/14728222.2014.980235. PMID:25491730 25491730

[12] Meng X, Huang Z, Teng F, Xing L, Yu J Predictive biomarkers in PD-1/PD-L1 checkpoint blockade immunotherapy. Cancer Treat Rev. 2015;41:868-76. doi:10.1016/j.ctrv.2015.11.001. PMID:26589760 26589760

[13] Kluger HM, Zito CR, Turcu G, Baine M, Zhang H, Adeniran A, Sznol M, Rimm DL, Kluger Y, Chen L et al. PD-L1 studies across tumor types, its differential expression and predictive value in patients treated with immune checkpoint inhibitors. Clin Cancer Res. 2017; 23(15):4270-4279. doi:10.1158/1078-0432.CCR-16-3146. PMID:28223273. [Epub 2017, Feb 21] 28223273 PMC5540774

[14] Reck M, Rodriguez-Abreu D, Robinson AG, Hui R, Csoszi T, Fulop A, Gottfried M, Peled N, Tafreshi A, Cuffe S et al. Pembrolizumab versus Chemotherapy for PD-L1-Positive Non-Small-Cell Lung Cancer. N Engl J Med. 2016;375:1823-33. doi:10.1056/NEJMoa1606774. PMID:27718847 27718847

[15] Rozali EN, Hato SV, Robinson BW, Lake RA, Lesterhuis WJ Programmed death ligand 2 in cancer-induced immune suppression. Clin Dev Immunol. 2012;2012:656340. doi:10.1155/2012/656340. PMID:22611421 22611421 PMC3350956

[16] Yearley JH, Gibson C, Yu N, Moon C, Murphy E, Juco J, Lunceford J, Cheng J, Chow LQM, Seiwert TY et al. PD-L2 Expression in Human Tumors: Relevance to Anti-PD-1 Therapy in Cancer. Clin Cancer Res. 2017;23:3158-67. doi:10.1158/1078-0432.CCR-16-1761. PMID:28619999 28619999

[17] Chen J, Jiang CC, Jin L, Zhang XD Regulation of PD-L1: a novel role of pro-survival signalling in cancer. Ann Oncol. 2016;27:409-16. doi:10.1093/annonc/mdv615. PMID:26681673 26681673

[18] Koh YW, Han JH, Park SY, Yoon DH, Suh C, Huh J GLUT1 as a Prognostic Factor for Classical Hodgkin's Lymphoma: Correlation with PD-L1 and PD-L2 Expression. J Pathol Transl Med. 2017;51:152-8. doi:10.4132/jptm.2016.11.03. PMID:28219001 28219001 PMC5357756

[19] Pinato DJ, Ramachandran R, Toussi ST, Vergine M, Ngo N, Sharma R et al. Immunohistochemical markers of the hypoxic response can identify malignancy in phaeochromocytomas and paragangliomas and optimize the detection of tumours with VHL germline mutations. Br J Cancer. 2013;108(2):429-37. doi:10.1038/bjc.2012.538. [Epub 2012, Dec 20] 23257898 PMC3566818

[20] Jochmanova I, Yang C, Zhuang Z, Pacak K Hypoxia-inducible factor signaling in pheochromocytoma: turning the rudder in the right direction. J Natl Cancer Inst. 2013;105(17):1270-83. doi:10.1093/jnci/djt201. PMID:23940289. [Epub 2013, Aug 12] 23940289 PMC3888279

[21] Pinato DJ, Tan TM, Toussi ST, Ramachandran R, Martin N, Meeran K, Ngo N, Dina R, Sharma R An expression signature of the angiogenic response in gastrointestinal neuroendocrine tumours: correlation with tumour phenotype and survival outcomes. Br J Cancer. 2014;110:115-22. doi:10.1038/bjc.2013.682. PMID:24231952 24231952 PMC3887289

[22] Pinato DJ, Shiner RJ, White SD, Black JR, Trivedi P, Stebbing J, Sharma R, Mauri FA Intra-tumoral heterogeneity in the expression of programmed-death (PD) ligands in isogeneic primary and metastatic lung cancer: Implications for immunotherapy. Oncoimmunology. 2016;5:e1213934. doi:10.1080/2162402X.2016.1213934. PMID:27757309 27757309 PMC5048760

[23] Cassol CA, Winer D, Liu W, Guo M, Ezzat S, Asa SL Tyrosine kinase receptors as molecular targets in pheochromocytomas and paragangliomas. Mod Pathol. 2014;27:1050-62. doi:10.1038/modpathol.2013.233. PMID:24390213 24390213 PMC4977182

[24] Dahia PL, Ross KN, Wright ME, Hayashida CY, Santagata S, Barontini M, Kung AL, Sanso G, Powers JF, Tischler AS et al. A HIF1alpha regulatory loop links hypoxia and mitochondrial signals in pheochromocytomas. PLoS Genet. 2005;1:72-80. doi:10.1371/journal.pgen.0010008. PMID:16103922 16103922 PMC1183527

[25] Pinato DJ, Ramachandran R, Toussi ST, Vergine M, Ngo N, Sharma R, Lloyd T, Meeran K, Palazzo F, Martin N et al. Immunohistochemical markers of the hypoxic response can identify malignancy in phaeochromocytomas and paragangliomas and optimize the detection of tumours with VHL germline mutations. Br J Cancer. 2013;108:429-37. doi:10.1038/bjc.2012.538. PMID:23257898 23257898 PMC3566818

[26] Noman MZ, Hasmim M, Messai Y, Terry S, Kieda C, Janji B, Chouaib S Hypoxia: a key player in antitumor immune response. A review in the theme: cellular responses to hypoxia. Am J Physiol Cell Physiol. 2015;309:C569-79. doi:10.1152/ajpcell.00207.2015. PMID:26310815 26310815 PMC4628936

[27] Koh YW, Han JH, Park SY, Yoon DH, Suh C, Huh J GLUT1 as a prognostic factor for classical hodgkin's lymphoma: correlation with PD-L1 and PD-L2 expression. J Pathol Transl Med. 2017;51:152-158. doi:10.4132/jptm.2016.11.03. PMID:28219001 28219001 PMC5357756

[28] Fishbein L, Leshchiner I, Walter V, Danilova L, Robertson AG, Johnson AR, Lichtenberg TM, Murray BA, Ghayee HK, Else T et al. Comprehensive molecular characterization of pheochromocytoma and paraganglioma. Cancer Cell. 2017;31:181-93. doi:10.1016/j.ccell.2017.01.001. PMID:28162975 28162975 PMC5643159

[29] Pollard PJ, El-Bahrawy M, Poulsom R, Elia G, Killick P, Kelly G, Hunt T, Jeffery R, Seedhar P, Barwell J et al. Expression of HIF-1alpha, HIF-2alpha (EPAS1), and their target genes in paraganglioma and pheochromocytoma with VHL and SDH mutations. J Clin Endocrinol Metab. 2006;91:4593-8. doi:10.1210/jc.2006-0920. PMID:16954163 16954163

