# Supplementary material for: Programmed cell death ligands expression in phaeochromocytomas and paragangliomas: Relationship with the hypoxic response, immune evasion and malignant behavior
Source: Oncoimmunology. 2017 Aug 4;6(11):e1358332. doi: 10.1080/2162402X.2017.1358332 (PMC5674959; doi:10.1080/2162402X.2017.1358332)
Supplement: Supplementary_materials.doc [file koni-06-11-1358332-s001.doc]

**Programmed cell death ligands expression in phaeochromocytomas and paragangliomas: relationship with the hypoxic response, immune evasion and malignant behaviour.**

Pinato D.J. et al.

**Online-only Supplement**

|  |  | **Page** |
| --- | --- | --- |
| **Figure S1** | Representative sections of PCC/PGL TMA cores. | 2 |
| **Figure S2** | Kaplan-Meier curves describing the overall survival of patients with PCC/PGL | 3 |
| **Table S1** | Prognostic factors for Overall Survival (OS) in PCC/PGL. | 4 |
| **Table S2** | Gene sets enriched in PD-L1 overexpressing PCC/PGL in positive correlation. | 5 |
| **Table S3** | Gene sets enriched in PD-L2 overexpressing PCC/PGL in positive correlation | 6 |

**Figure S1.** Representative sections of PCC/PGL TMA cores immunostained for CaIX (**Panel A**), Hif-1α (**Panel B**) VEGF-A (**Panel C**). **Panel D** shows a negative immunohistochemical reaction.


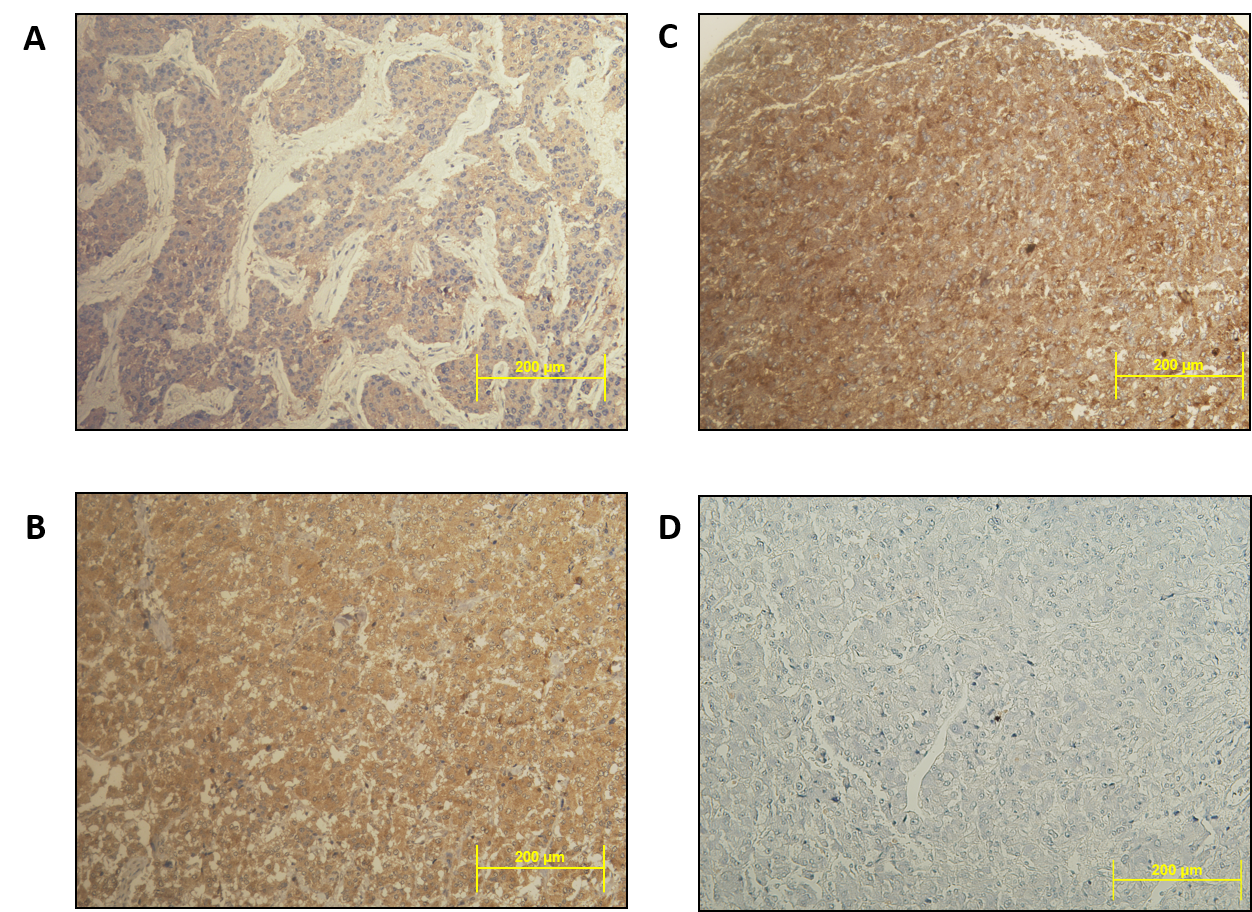


**Figure S2.** Kaplan-Meier curves describing the overall survival of patients with PCC/PGL with Vascular Invasion (**Panel A**), Necrosis (**Panel B**) and Capsular Invasion (**Panel C**) emerging as prognostic factors.

**
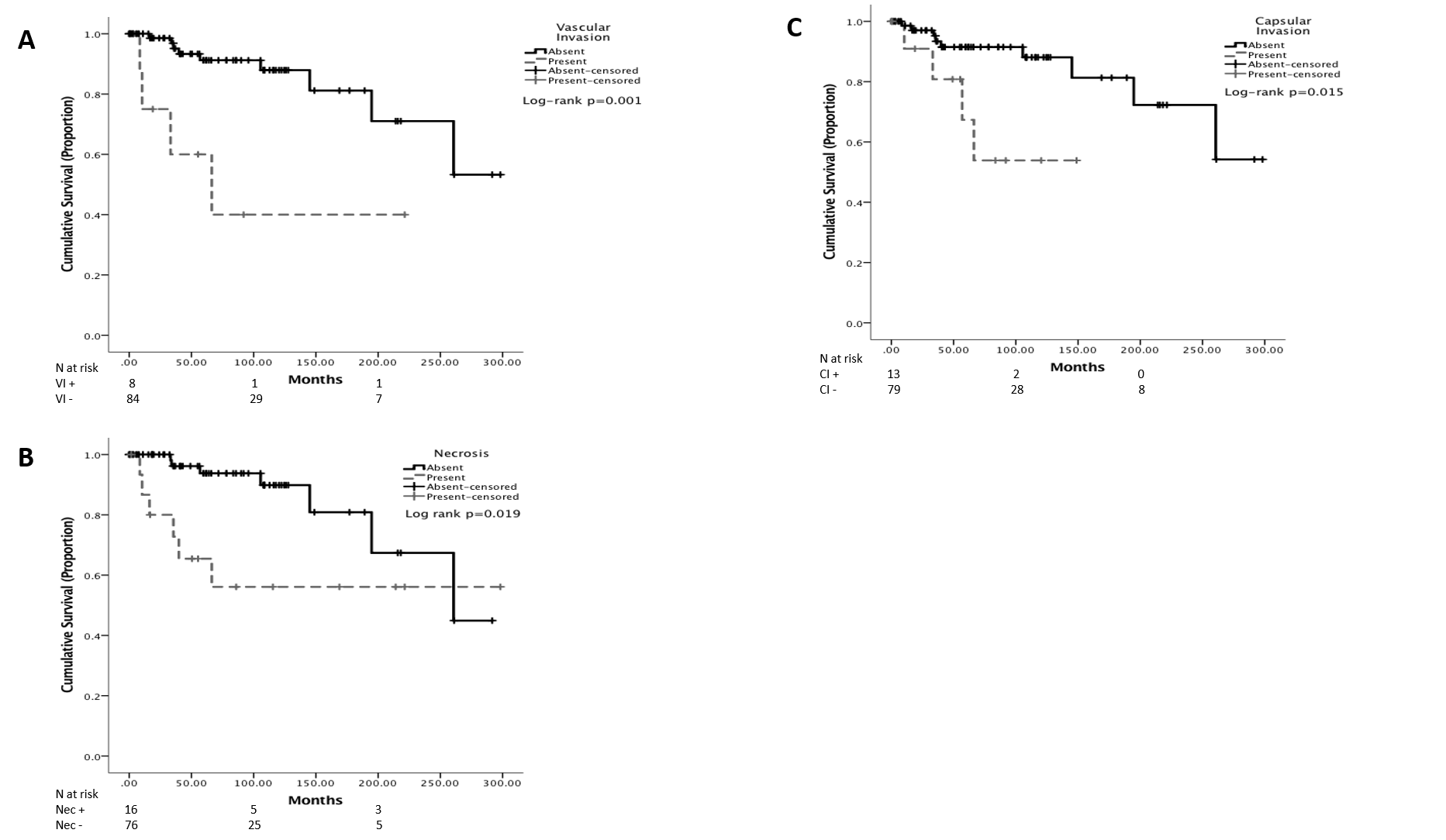
**

**Table S1.** Prognostic factors for Overall Survival (OS) in PCC/PGL.

| **Variable** | | **Mean OS in months (standard error)** | **Hazard Ratio**  **95% CI** | **p value** |
| --- | --- | --- | --- | --- |
| **PD-L1 expression** | Positive (18)  Negative (82) | 260 (-)  280 (±34) | - | 0.10 |
| **PD-L2 expression** | Positive (16)  Negative (84) | 162 (±31)  309 (±33) | 3.1 (1.0-9.2) | **0.029** |
| **Vascular Invasion** | Present (8)  Absent (84) | 109 (±37)  241 (±17) | 6.2 (1.8-20) | **<0.001** |
| **Capsular Invasion** | Present (13)  Absent (79) | 242 (±16)  101 (±18) | 4.1 (1.2-14.2) | **0.015** |
| **Necrosis** | Present (16)  Absent (76) | 181 (±36)  235 (±18) | 3.4 (1.1-10.4) | **0.027** |

| **Table S2.**  Gene sets enriched in PD-L1 overexpressing PCC/PGL in positive correlation. | | | | | | |
| --- | --- | --- | --- | --- | --- | --- |
| **Gene Set** | **Size** | **ES** | **NES** | **Nominal**  **p-value** | **FDR**  **q-value** | **FWER p-value** |
| HALLMARK_PROTEIN_SECRETION | 96 | 0.51 | 1.70 | 0.03 | 1 | 0.519 |
| HALLMARK_INTERFERON_ALPHA_RESPONSE | 97 | 0.59 | 1.60 | 0.12 | 1 | 0.636 |
| HALLMARK_INTERFERON_GAMMA_RESPONSE | 199 | 0.52 | 1.58 | 0.14 | 1 | 0.669 |
| HALLMARK_IL6_JAK_STAT3_SIGNALING | 84 | 0.48 | 1.56 | 0.15 | 0.98 | 0.695 |
| HALLMARK_INFLAMMATORY_RESPONSE | 197 | 0.38 | 1.33 | 0.24 | 0.74 | 0.91 |
| HALLMARK_IL2_STAT5_SIGNALING | 197 | 0.31 | 1.14 | 0.24 | 0.78 | 0.98 |
| HALLMARK_TGF_BETA_SIGNALING | 54 | 0.33 | 1.06 | 0.33 | 0.75 | 1.00 |
| HALLMARK_TNFA_SIGNALING_VIA_NFKB | 199 | 0.32 | 0.94 | 0.43 | 0.76 | 1.00 |
| HALLMARK_HYPOXIA | 197 | 0.24 | 0.83 | 0.47 | 0.73 | 1.00 |

| **Table S3.**  Gene sets enriched in PD-L2 overexpressing PCC/PGL in positive correlation. | | | | | | |
| --- | --- | --- | --- | --- | --- | --- |
| **Gene Set** | **Size** | **ES** | **NES** | **Nominal**  **p-value** | **FDR**  **q-value** | **FWER p-value** |
| HALLMARK_INFLAMMATORY_RESPONSE | 197 | 0.80 | 2.71 | <0.001 | 0.00 | <0.001 |
| HALLMARK_IL6_JAK_STAT3_SIGNALING | 84 | 0.81 | 2.63 | <0.001 | 0.00 | <0.001 |
| HALLMARK_INTERFERON_GAMMA_RESPONSE | 199 | 0.80 | 2.43 | <0.001 | 0.00 | 0.01 |
| HALLMARK_IL2_STAT5_SIGNALING | 197 | 0.68 | 2.40 | <0.001 | 0.00 | 0.01 |
| HALLMARK_TNFA_SIGNALING_VIA_NFKB | 199 | 0.79 | 2.29 | <0.001 | 0.00 | 0.02 |
| HALLMARK_HYPOXIA | 197 | 0.68 | 2.23 | <0.001 | 0.01 | 0.03 |
| HALLMARK_INTERFERON_ALPHA_RESPONSE | 97 | 0.80 | 2.20 | <0.001 | 0.01 | 0.04 |
| HALLMARK_TGF_BETA_SIGNALING | 54 | 0.65 | 2.00 | 0.01 | 0.03 | 0.16 |
